# Antibacterial and antibiofilm activities of star anise-cinnamon essential oil against multidrug-resistant *Salmonella* Thompson

**DOI:** 10.3389/fcimb.2024.1463551

**Published:** 2025-03-03

**Authors:** Jie Zhang, Dapei Zhang, Yanhua Chen, Yongyu Gong, Binfang Yuan, Zhiyuan Mo, Haibo Tang, Junyu Tao, Ziheng Xu

**Affiliations:** ^1^ Guangxi Scientific Research Center of Traditional Chinese Medicine, Guangxi University of Chinese Medicine, Nanning, Guangxi, China; ^2^ Guangxi Key Laboratory of Translational Medicine for Treating High-Incidence Infectious Diseases with Integrative Medicine, Institute of Traditional Chinese and Zhuang-Yao Ethnic Medicine, Guangxi University of Chinese Medicine, Nanning, Guangxi, China; ^3^ School of Public Health and Management, Guangxi University of Chinese Medicine, Nanning, Guangxi, China

**Keywords:** *Salmonella* Thompson, star anise-cinnamon essential oil, antibacterial, anti-biofilm, *Bellamya quadrata*

## Abstract

**Introduction:**

The emergence of foodborne multidrug-resistant (MDR) *Salmonella* has attracted considerable global attention. Given that food is the primary transmission route, our study focuses on *Bellamya quadrata*, a freshwater snail that is commonly consumed as a specialty food in Guangxi, China.

**Methods:**

Eight MDR *Salmonella* strains were isolated from *Bellamya quadrata* samples collected across various markets. Previous animal experiments have confirmed their lethality in mice. We determined the minimum inhibitory concentrations (MICs) and fractional inhibitory concentration (FIC) indices of cinnamon essential oil (CEO) and star anise essential oil (SAEO) using the microdilution plate and checkerboard methods. The time-kill curve method was employed to assess the antibacterial activity of the cinnamon-star anise essential oil (SCEO) against planktonic MDR *Salmonella*. The alkaline phosphatase assay and fluorescence microscopy demonstrated that SCEO causes damage to bacterial cell walls and membranes. Crystal violet staining and scanning electron microscopy (SEM) were used to observe changes in biofilms after SCEO treatment. Quantitative real-time PCR was utilized to analyze the expression of genes related to biofilm formation following SCEO treatment.

**Results:**

The MIC of SAEO was determined to be 25 mg/mL, whereas that of CEO was significantly lower at 0.62 mg/mL. The FIC index calculated was 0.375, which suggests a synergistic interaction between the two. When SCEO was used in combination at specific ratios, it demonstrated enhanced antibacterial and anti-biofilm capabilities compared to the individual effects of CEO or SAEO, potentially through the disruption of bacterial cell membranes and cell walls. However, in *Salmonella* treated with SCEO, an upregulation in the expression of biofilm-associated genes was observed, including *csgA, adrA, bcsA*, and *csgD*. This increase may be attributed to stress-induced transcriptional responses within the bacteria.

**Discussion:**

SCEO significantly impacts cell wall integrity, suggesting its crucial role in reducing biofilm formation. These findings indicate that SCEO holds potential as an alternative to traditional antibiotics and merits further scientific investigation and development.

## 1 Introduction

*Salmonella* represents a significant threat to global public health as an important zoonotic disease pathogen. According to the World Health Organization (2015), it is estimated that contaminated food causes up to 600 million illnesses each year. Approximately 350 million of these illnesses are attributed to pathogenic bacteria. *Salmonella* is one such pathogen responsible for these bacterial illnesses. The issue of food-borne *Salmonella* is becoming increasingly prevalent. The infection of *Salmonella* from food to humans is considered to be the primary route of transmission. Food-borne *Salmonella* infections make up a large percentage of all food-related illnesses. This is especially true during summer, which is the peak season for food-borne poisoning. Consumption of contaminated fruits, vegetables, meat, seafood, and other foods is one of the primary routes of *Salmonella* infection (Akil et al., 2014). The wide range of hosts for *Salmonella* includes domestic animals such as poultry, cattle and pigs, as well as wildlife, pets, fish and rodents. Furthermore, the existence of asymptomatic infected animals, which spread the *Salmonella* pathogen via feces, complicates pathogen control. This is because *Salmonella* can persist in contaminating crops, particularly vegetables and fruits, through soil and water (Chlebicz and Śliżewska, 2018). Previously, our team successfully isolated eight distinct strains of *Salmonella* Thompson from *Bellamya quadrata* collected in Guangxi, China. *Bellamya quadrata* are a traditional delicacy in South China, yet they are frequently contaminated with *Salmonella*, a significant hazard to human life and health. Previously, our team confirmed the pathogenicity of *Salmonella* Thompson obtained from food *Bellamya quadrata* isolated from Guangxi, China. These were found to cause liver, spleen, and kidney morbidity in Kunming mice (**Supplementary Figures S1A-C**: Blank Group; **Supplementary Figure S1E, F**: infection group with C6304; **Supplementary Figure S2A, B**: Shows changes in liver and intestinal microbiota before and after), with mortality rates reaching 100%. This evidence demonstrates the serious public health threat posed by these *Salmonella* Thompson. Therefore, the prevention and control of foodborne *Salmonella* is of paramount importance.

Currently, the clinical defense and control of *Salmonella* involves the use of antibiotics. However, the extensive use of antibiotics has resulted in the development of drug resistance in *Salmonella*, with multi-drug resistant *Salmonella* being increasingly reported in recent years. The resistance mechanism of *Salmonella* has also been widely concerned. One of the group resistance mechanisms of severely resistant bacteria is biofilm (BF).The colonization of *Salmonella* under natural conditions is dependent on the formation of a bacterial BF on its surface. *Salmonella* responds to damage to the organism from external environmental factors by forming BF (Jahan et al., 2022). Bacteria that form BFs exhibit up to 1,000 times greater antibiotic resistance compared to when they are in their suspended state (Mah, 2012). BF is a three-dimensional microbial community (Jiang et al., 2021). Bacteria, enveloped in a membrane-like matrix, adhere to the surfaces of both living and inanimate objects (Das et al., 2013). This matrix is comprised of bacterially secreted Extracellular Polymeric Substances (EPS), which include extracellular polysaccharides and proteins. These substances EPS allow the bacteria to form highly organized communities. The formation of BF provides *Salmonella* with a protective barrier against host immune responses and unfavorable factors in the environment, such as physical or chemical (MacKenzie et al., 2017). Once a *Salmonella* BF has formed during food processing or storage, it is challenging to eradicate using conventional cleaning and disinfection methods, which may result in persistent contamination of food and outbreaks of foodborne illnesses (Pang et al., 2023). Consequently, the capacity to eliminate and remove the BF from the surface of *Salmonella* represents a pivotal aspect in the search for novel and efficacious biocides.

Natural plant essential oils have become a subject of intense research interest due to their potential antimicrobial properties. Cinnamomum cassia (*Cinnamomum cassia*(L.) D. Don) is one of the ten varieties geo-authentic traditional Chinese medicine of Guangxi. Cinnamon essential oil (CEO) is a volatile oil extracted from the bark, leaves or flower buds of Cinnamomum cassia, the main components of which include cinnamaldehyde, eugenol, etc. These compounds have been demonstrated to exhibit potent antimicrobial activity against Escherichia coli, among other microorganisms (Raeisi et al., 2015). Star anise (*Illicium verum* Hook. f.), one of the ten varieties geo-authentic traditional Chinese medicine of Guangxi, is distributed in Guangxi, Guangdong, Guizhou and Yunnan in China. SAEO is extracted from star anise fruits, and its main components are anethole and anisaldehyde, which also have significant antibacterial properties (Luís et al., 2019). Although the antibacterial activity of SAEO and CEO against *Salmonella* was confirmed *in vitro*. However, the information of the inhibitory effect and mechanism of Star Anise-Cinnamon Essential Oil (SAEO and CEO combined essential oil, SCEO) on *Salmonella* and its BF is very limited. SCEO not only contains components like cinnamaldehyde and eugenol from cinnamon oil, but also incorporates active substances such as anethole and anisaldehyde from star anise oil. The synergistic effect of these components may endow SCEO with enhanced antibacterial and anti-BF activities. The objective of this study was to assess the mechanism of antibacterial and anti-BF activities of SCEO against on MDR *Salmonella* Thompson isolated from the *Bellamya quadrata*.

## 2 Materials and methods

### 2.1 Extraction of CEO and SAEO

The CEO and SAEO was extracted and obtained by Dr. Xu Ziheng’s group at Guangxi University of Chinese Medicine. The accuracy and reliability of the method were verified by Associate Professor Tao Junyu at the same institution. The primary reference methods (Haro-González et al., 2021; Ellboudy et al., 2023) for the extraction process are outlined as follows:

Cinnamon bark was crushed in a pulverizer (Model FW177, Tianjin, China) and sieved through a No. 4 sieve. Subsequently, 100 grams of the powdered cinnamon bark were mixed with 700 mL of water. The essential oil-water mixture was obtained via steam distillation. After 2 hours of heating, sodium chloride powder (supplied by Beijing Solarbio Science & Technology Co., Ltd., China) was added to the mixture to achieve a final concentration of 0.1 mg/mL. The mixture was then thoroughly mixed and poured into a separator for overnight static separation. The lower aqueous layer was discarded, while the yellowish upper layer was retained and placed in a sealed bottle containing anhydrous sodium sulfate (Beijing Solarbio Science & Technology Co., Ltd., China) for an overnight static period. Thereafter, the upper layer was transferred to a new bottle for light-avoiding storage at room temperature.

For the extraction of SAEO, the anise fruit was subjected to a crushing process using a No. 2 sieve. Subsequently, 50 grams of anise powder were combined with 500 mL of water and subjected to steam distillation for a period of 2 hours, during which time the essential oil and water mixture was formed. The subsequent processing steps were identical to those employed in the extraction of CEO.

### 2.2 Bacterial strains and culture conditions

Eight strains were obtained from the Guangxi University of Chinese Medicine in China. These strains were derived from *Bellamya quadrata* sold in various markets and identified as *Salmonella* Thompson by 16sRNA sequencing.

### 2.3 Identification of the ability of the BF formation of *Salmonella*

The strains were activated in the sterilized Luria-Bertani (LB) (Beijing Solarbio Science & Technology Co., Ltd., China) liquid medium through incubation on a constant temperature shaker (Shanghai Yuejin Medical Equipment Co., Ltd.) at 150 rpm and 37°C for 24 hours. And a crystal violet staining method (O'Toole and Kolter, 1998) was used to identify the strains with strong BF formation ability (Ommen et al., 2017).

The activated bacterial solution was inoculated into 96-well microtiter plates (Labgic Technology Co., Ltd., China) with a final concentration of 1×10^6^ CFU/mL and then incubated for 48 hours until the BF attained maturity. At first, most of the bacterial solution was decanted and washed twice with phosphate-buffered saline (PBS) (Labgic Technology Co., Ltd., China) to remove non-adherent cells. Subsequently, methanol (Chengdu Kelong Chemical Co., Ltd., China) was added for fixation for 15 minutes, after which the methanol was discarded and allowed to evaporate completely. Then, crystal violet staining was conducted for a period of five minutes, after which the crystal violet was discarded. After being washed with clear water until the effluent was colorless, anhydrous ethanol (Chengdu Kelong Chemical Co., Ltd., China) was added for decolorization. Finally, the absorbance was quantified at 570 nm with a microplate reader (Experiments were conducted using the INFINITE 200 PRO instrument made by Tecan Spark, Austria).

In brief, the mean optical density (OD) value of the negative control group was recorded as ODc (Atshan et al., 2012; Knobloch et al., 2002). BF formation ability was classified in the following ways: strong BF production (4 ODc < OD), moderate BF production (2ODc < OD ≤ 4ODc), weak BF production (ODc < OD ≤ 2ODc), and no BF production (OD ≤ ODc). The classification criteria were based on previous studies (Christensen et al., 1985; Stepanović et al., 2000) and were chosen to facilitate comparison with other BF formation bacteria.

In this study, the Salmonella C6304 exhibiting the highest OD values within the strong BF production group were selected for subsequent experiments.

### 2.4 phenotypic detection of bacterial antibiotic resistance using disk diffusion method

A colony was selected from a freshly cultured bacterial plate and inoculated into LB broth for overnight incubation at 37°C. The bacterial suspension was then diluted with PBS to achieve a concentration of 1×10^8^ CFU/mL. Using a sterile cotton swab, the suspension was evenly spread onto MH agar plates. Sterile forceps were subsequently used to carefully place commercial antibiotic disks (Changde BKMAM Biotechnology Co., Ltd., China) containing predetermined concentrations of antimicrobial agents, onto the surface of the agar plates. Each plate received four distinct antibiotic disks, and this arrangement was replicated three times. The inoculated plates were then incubated at 37°C for 24 hours. After the incubation period, a vernier caliper was used to measure the diameters of the resulting inhibition zones. These measurements were analyzed in accordance with CLSI M100 (CLSI, 2021), which classify bacterial susceptibility to the respective antibiotics as sensitive (S), intermediate (I), or resistant (R). To ensure experimental accuracy, *Escherichia coli* (ATCC^®^ 25922™) and *Staphylococcus aureus* (ATCC^®^ 25923™) were used as quality control strains alongside the test strains in the antimicrobial sensitivity testing, validating the reliability of our experimental conditions and methodologies (**Supplementary Table S1**).

### 2.5 Assessment of the MIC of SCEO against *Salmonella*

In this study, we used a special MIC testing method to avoid interference from the inherent color of the essential oils and minimize human error in MIC determination. This method was mainly based on reference (Ksouda et al., 2019), with certain modifications made. The details of the modified MIC test method are as follows:

Tween-80 (Beijing Solarbio Science & Technology Co., Ltd., China) was incorporated into the MH broth medium at a dilution of 1%, as specified. A volume of 100 μL of CEO was added to a 10-mL dilution solution to achieve an initial concentration of 10 μL/mL. This mixture was then thoroughly homogenized. A total volume of 500 μL of the dilution solution was transferred to an EP tube (Beijing Labgic Technology Co., Ltd. China), and the master batch of CEO was serially diluted to concentrations of 10 μL/mL, 5 μL/mL, 2.5 μL/mL, 1.25 μL/mL, 0.62 μL/mL, 0.31 μL/mL, 0.16 μL/mL, and 0.08 μL/mL using the gradient dilution method. The dilution solution served as a blank. The SAEO was diluted using the same methodology described for the CEO, achieving final concentrations of 100 μL/mL, 50 μL/mL, 25 μL/mL, 12.5 μL/mL, 6.25 μL/mL, 3.12 μL/mL, 1.56 μL/mL, and 0.78 μL/mL.Additionally, the MIC of levofloxacin was tested at concentrations of 10 μg/mL, 5 μg/mL, 2.5 μg/mL, 1.25 μg/mL, 0.625 μg/mL, 0.312 μg/mL, 0.156 μg/mL, 0.078 μg/mL, 0.039 μg/mL.*Escherichia coli* (ATCC^®^ 25922™) and *Staphylococcus aureus* (ATCC^®^ 25923™) were used as quality control strains.

The test bacterial solution was diluted to 1×10^7^ CFU/mL, and then 50 μL was inoculated into EP tubes containing different drug concentrations. The tubes were incubated in a constant temperature shaking incubator at 37 ± 1°C at a speed of 150 rpm for 20 hours.

The detection protocol involved using a pipette gun to transfer 180 μL from each EP tube into 96-well plates, followed by the addition of 20 μL of a diluted 0.1% red tetrazolium salt solution (Beijing Solarbio Science & Technology Co., Ltd., China) to each well. The plates were then incubated for 4 hours. After this incubation period, the absorbance at 485 nm was measured by the microplate reader (INFINITE 200 PRO instrument made by Tecan Spark, Austria). Alternatively, the lowest concentration of drug solution at which the bacterial solution did not turn red was visually determined as the MIC.

### 2.6 Determination of the fractional inhibitory concentration of SAEO and CEO

To assess the combination effect of the two essential oils, a checkerboard method was employed. Specifically, 300 µL of each oil was pipetted into individual EP tubes in a systematic pattern.

Salmonella cultures were first incubated overnight and subsequently diluted 100-fold. Following this, 50 µL of the diluted culture was added to each EP tube containing the essential oil combinations. The tubes were then incubated for 20 hours at a constant temperature of 37°C in a shaking incubator set at 150 rpm.

After incubation, 180 µL from each EP tube was transferred to a 96-well plate. To each well, 20 µL of a 0.1% red tetrazolium saline solution (Beijing Solarbio Science & Technology Co., Ltd., China) was added. Following a four-hour incubation period, absorbance was measured at 485 nm using an enzyme marker (INFINITE 200 PRO, Tecan Spark, Austria). The MIC value was determined as the lowest concentration of the essential oil solution that did not result in a red coloration indicative of bacterial growth. Subsequently, the fractional inhibitory concentration (FIC) index was calculated (Xu et al., 2018; Requena et al., 2019).The FIC index is used to evaluate the effect of drug combinations. An FIC value less than or equal to 0.5 indicates synergism, a value greater than 0.5 but less than or equal to 4 indicates additivity, and a value greater than 4 indicates antagonism.


$$\text{FIC}={\text{MIC}}_{\text{A}\_\text{comb}}/{\text{MIC}}_{\text{A}\_\text{alone}}+{\text{MIC}}_{\text{B}\_\text{comb}}/{\text{MIC}}_{\text{B}\_\text{alone}}$$

### 2.7 SCEO time-kill curve test measurements

To prepare the broth dilutions, Tween 80 was added to sterilized MH broth at a concentration of 1%, followed by ultrasonic vortexing to ensure a homogeneous solution, crucial for proper dispersion during experiments. Subsequently, the CEO and SAEO were sequentially diluted to sub-MIC levels, with the initial concentration set as the starting point. Composite blends of these essential oils were also individually diluted to sub-synergistic concentrations (refer to results in 2.5). A gradient volume of 480 μL of the essential oil-containing solution was then added to Eppendorf tubes, followed by the addition of 20 μL of bacterial suspension to each tube, resulting in an initial bacterial concentration of approximately 1×10^6^ CFU/mL. The tubes were incubated at 37°C to evaluate the antibacterial activity. For comparison, a blank control (Tween-containing medium without essential oils) and a positive control (bacterial suspension without essential oils) were also included and incubated under the same conditions for various time points (1, 3, 6, 10, and 24 hours). Colony counts were performed at each time point to quantify bacterial viability, and a time-kill curve was constructed by plotting the mean colony count (log10 CFU/mL) against time, providing insight into the antibacterial efficacy of the essential oils over the course of the incubation.

### 2.8 *In vitro* inhibitory effect of SCEO on *Salmonella* BF

To evaluate the antibacterial and anti-biofilm activities of the CEO and SAEO, broth dilutions were first prepared by adding Tween 80 to sterilized MH broth at a 1% concentration and then ultrasonically vortexing for homogeneity. The CEO and SAEO were sequentially diluted to sub-MIC levels, with the initial highest concentration serving as the starting point to assess their individual antibacterial activities at reduced concentrations. Additionally, composite essential oil blends were configured to sub-synergistic concentrations to evaluate their combined effects. A volume of 180 μL of the drug solution was dispensed in a gradient manner into each well of a 96-well plate, followed by the addition of bacterial solution to achieve an initial concentration of 1×10^6^ CFU/mL. Sterilized polypropylene screws were placed in each well and incubated at 37°C for 48 hours to assess bacterial adhesion and biofilm formation on a non-porous surface. After incubation, the screws were washed and stained with crystal violet to visualize adherent bacteria. The bound crystal violet was eluted with anhydrous ethanol, and the absorbance of the eluted solution was measured at 570 nm to quantify biofilm formation. This standardized method, similar to those employed in previous studies, enabled precise and reproducible quantification of bacterial biofilm on the screws, providing insights into the antibacterial and anti-biofilm properties of the tested essential oils.

This method allowed for the precise and reproducible quantification of bacterial BF formation on a standardized surface, providing valuable insights into the antibacterial and anti-BF activities of the tested essential oils. Similar methods have been used in previous studies to assess the efficacy of various antibacterial agents against BF-forming bacteria (Brożyna et al., 2022).

### 2.9 SEM to determine the effect of SCEO *in vitro* on the morphology of suspended MDR *Salmonella* organisms

Drawing upon Junyu Tao’s work (Tao et al., 2021) with some methodological optimizations, the experimental procedures were as follows. For the preparation of broth dilution, Tween 80 was added to sterilized MH broth at a 1% ratio to facilitate the dispersion of the essential oils. In the preparation of the essential oil emulsion, two essential oils were sequentially diluted to sub-MIC levels, with the initial highest concentration as the starting point.

For the addition method, a volume of 1 mL of the drug solution was introduced into each EP tube according to a predetermined gradient. Subsequently, the bacterial solution was added to achieve an initial bacterial concentration of 1×10^6^ CFU/mL, ensuring consistent and precise dosing. After 9 hours of incubation, the bacterial suspension was centrifuged at 12000 rpm for 2 minutes (5424R, Eppendorf AG,German) to separate the bacterial cells from the broth. The supernatant was discarded, and the bacterial pellet was fixed in 2.5% formaldehyde electron microscope fixative at 4°C overnight.

The following day, the bacterial cells were washed with a series of ethanol solutions (40%, 60%, 80%, 90%, 95%, and 100%) to remove residual fixative and dehydrate the cells. After dehydration, the ethanol was replaced with ethyl acetate, and the bacterial cells were allowed to air-dry, resulting in a bacterial powder. This powder was then sprayed with gold (ISC 150 Ion Sputter Soater, supro instruments co. Ltd,China) to enhance conductivity and facilitate observation under an electron microscope (Phenom XL,Thermo Fisher Scientific, America).The observation conditions are set as follows: acceleration voltage of 5kv, beam current intensity in point mode, and probe mode in mixed mode.

### 2.10 SEM to determine the effect of SCEO *in vitro* on the morphology of adherent *Salmonella* MDR organisms

Drawing inspiration from the methodological framework outlined (Tao et al., 2021), with subsequent optimizations, we devised the following experimental protocol. In a 6-well cell culture plate, 2 mL of drug solution (Same as in Step 2.8), containing essential oils diluted to sub-MIC or sub-synergistic concentrations was dispensed into each well according to a pre-established gradient. A calibrated volume of bacterial suspension was then inoculated into each well to achieve an initial bacterial concentration of 1×10^6^ CFU/mL. Round coverslips (25mm,biosharp therapeutics co. Ltd,China) were introduced as substrates for bacterial adherence and proliferation, and the plate was incubated under optimal conditions for 24 hours.

Upon completion of incubation, the round coverslips were gently extracted and subjected to a rigorous washing procedure, involving sequential immersion in ethanol solutions of increasing concentrations (40%, 60%, 80%, 90%, 95%, and 100%), with 10-minute intervals between each step, to eliminate residual culture media and fixatives. This was followed by two rinses with ethyl acetate and subsequent freeze-drying to preserve the intricate morphological features of the adherent bacteria. This round coverslips was then sprayed with gold (ISC 150 Ion Sputter Soater, supro instruments co. Ltd,China) to enhance conductivity and facilitate observation under an electron microscope (Phenom XL,Thermo Fisher Scientific, America).The observation conditions are set as follows: acceleration voltage of 5 kV, beam current intensity in point mode, and probe mode in mixed mode.

To quantitatively analyze the inhibition of bacterial adhesion following drug treatment, we employed ImageJ software (version 1.8.0_345) for processing the collected SEM images. In this study, we leveraged ImageJ’s automatic measurement to accurately calculate the area of bacterial clusters in 6 randomly selected fields of view at 1000× magnification for different drug treatment groups.

### 2.11 RT-qPCR assay for the detection of BF-associated gene expression

Log phase *Salmonella* cells (10^8^ CFU/mL) were added to 6-well microtiter plates after treatment with or without successive concentrations of CEO, SAEO and SCEO at 1/2 MIC concentration. The suspension was centrifuged at 5000 × g for 1 min and washed with DEPC-treated water. Cell lysates were collected and total RNA was isolated with TRIzol and then treated with DNAse. The cDNA template was reverse transcribed from the RNA using a Thermo Fisher Scientific Reverse Transcription Kit. The expression levels of target genes in *Salmonella* were determined by a fluorescence quantitative PCR (qPCR) assay as previously described. Expression values were calculated using the ΔΔCt method and expressed as fold change relative to control samples. gyrB was used as a housekeeping gene. All primers used in this study are listed in **Table 1**.

**Table 1:** qPCR sequences.

	Gene name		Sequences	Reference
1	*gyrB*	F	ACGCGTCTGTTGACCTTCTTC	(Chen et al., 2021)
2	*gyrB*	R	CTGTTCCTGCTTACCTTTCTTCAC	
3	*csgD*	F	CGGCCGGTTGCATTGTTTTA	
4	*csgD*	R	CCACGTGTTCCTGGTCTTCA	
5	*csgA*	F	TCGACCAGTGGAACGCTAAAA	
6	*csgA*	R	ACCAACCTGACGCACCATTAC	
7	*adrA*	F	GGCCATTAAATTAGCGGAAC	
8	*adrA*	R	AATAAAATTTCCCAGTGGCG	
9	*bcsA*	F	CGGGCGTGAATCATTTCGTC	
10	*bcsA*	R	TCAGGAACCAGCCCATTGTC	

The qPCR assay was performed with an initial denaturation step at 95°C for 3 minutes, followed by 40 cycles of amplification. Each amplification cycle consisted of denaturation at 95°C for 15 seconds, annealing at a temperature 55°C for 30 seconds, and extension at 72°C for 45 seconds. Following amplification, a melting curve analysis was conducted by gradually increasing the temperature from 60°C to 95°C, with fluorescence measurements taken at each 0.5°C increment to assess the specificity of the PCR products.

### 2.12 Detection of bacterial cell wall damage using an alkaline phosphatase assay kit

*Salmonella* was cultivated to the logarithmic growth phase. SAEO, CEO, SCEO and Levofloxacin—were subsequently diluted in MH broth to concentrations of MIC, 1/2MIC, and 1/4 MIC. To each EP tube containing 500 μL of diluted oil, 20 μL of *Salmonella* suspension was added, achieving a final concentration of 1 × 10^8^ CFU/mL. Following a 3-hour incubation at 37°C, the samples were centrifuged at 5000 rpm for 10 minutes, yielding supernatants.

For analysis, supernatants were processed per the alkaline phosphatase detection kit instructions (Beyotime Biotechnology, China). A blank control consisted of untreated but diluted *Salmonella*. Into a 96-well plate, 50 μL of supernatant was dispensed, followed by 50 μL of working solution. After 30 minutes, 100 μL of stop solution was added. Absorbance was measured at 425 nm, where darker reaction products indicated higher ALP activity. This procedure was triplicated for accuracy and reliability, with final results calculated as the average of measurements.

### 2.13 Membrane integrity

*Salmonella* was inoculated at a concentration of 1×10^6^ CFU/mL into a 6-well cell culture plate and incubated at 37°C for 24 hours. After removing the culture medium, SCEO was prepared in MH broth at concentrations of 1/4 MIC and 1/2 MIC and added to the wells for 3 hours, with blank MH broth as the control group. After 3 hours, the culture medium was removed, and the cells were washed twice with PBS. The FilmTracer™ Live/Dead Biofilm Viability Kit (Invitrogen, Thermo Fisher Scientific) was used to stain the cells, with PI (490/635 nm) and Syt9 (482/500 nm) added at working concentrations. The cells were incubated for 30 minutes, and fluorescence images were then captured using a Zeiss LSM 900 laser confocal microscope.

### 2.14 Statistical analysis

Data were analyzed using GraphPad Prism version 10.2 (GraphPad Software, Inc., La Jolla, CA, USA) for statistical calculations and graphical representations. Various statistical tests, including t-tests for pairwise comparisons and one-way analysis of variance (ANOVA) for multiple group comparisons, were performed as appropriate. The significance levels of P-values are denoted as follows: **** (P < 0.0001), *** (P < 0.001), ** (P < 0.01), and * (P < 0.05) indicate extremely highly significant, highly significant, significant, and somewhat significant differences, respectively. Conversely, a P-value greater than 0.05 (P > 0.05) suggests no significant difference.

For image assembly, Adobe Photoshop (Adobe Systems Incorporated, San Jose, CA, USA) was used to merge and adjust figure layouts without altering the integrity of the original data. Photoshop was strictly limited to non-quantitative adjustments, such as cropping, resizing, and minor color correction, to maintain consistency across all figures. Fluorescence microscopy images were processed using ZEN 3.8 (Carl Zeiss AG, Oberkochen, Germany) for image enhancement and analysis. The software facilitated adjustments to brightness, contrast, and other parameters to improve image clarity without affecting data interpretation. For scanning electron microscopy (SEM) image quantification, ImageJ (National Institutes of Health, Bethesda, MD, USA) was used.

## 3 Results

### 3.1 *Salmonella* MDR isolated from *Bellamya quadrata* has been demonstrated to possess a high capacity to form BFs

To thoroughly evaluate its BF-forming ability, we employed the crystal violet staining method (The results after staining are shown in **Supplementary Figure S3**) for detection according to the data in **Table 2**. The results indicate that all six bacterial strains tested exhibit robust BF-forming capabilities in **Table 3**, the calculation result for *Salmonella* (C6304) is 2.56, which is significantly greater than 4 times the ODc value (ODc = 0.53). Therefore, it is identified as a strong BF-forming strain. Given its unique BF-forming characteristics, this strain has been selected as the core research subject for all subsequent experiments.

**Table 2:** The crystal violet staining method for the screening results of strong film-forming MDR *Salmonella*.

Salmonella	C6272	C6273	C6277	C6304	C6458	C6465	C6469	C42	Blank
ODvalue	0.45	0.36	1.87	2.56	1.4	1.67	1.83	1.95	0.53

**Table 3:** Statistical table of *Salmonella* BF formation capacity.

BF forming capacity	Standard of judgement	Number of strains, n=8
Strong BF production	4ODc < OD	1 (12.5%)
Moderate BF production	2ODc < OD ≤ 4ODc	5 (62.5%)
Weak BF production	ODc < OD ≤ 2ODc	0 (0)
No BF production	OD ≤ ODc	2 (25%)

### 3.2 *Salmonella* MDR phenotype results

Based on **Table 4**, the drug sensitivity test results indicate that *Salmonella* C6304 exhibits varying degrees of susceptibility to different classes of antibiotics. Specifically, the strain is sensitive to beta-lactam antibiotics such as ampicillin and cefazolin, as well as to aminoglycosides like amikacin and gentamicin. However, it demonstrates resistance to antibiotics within the same class, including oxacillin and piperacillin. Among fluoroquinolones, ciprofloxacin and norfloxacin show sensitivity, while levofloxacin falls into the intermediate category. Regarding macrolides, the strain is only sensitive to azithromycin and resistant to erythromycin. Additionally, imipenem, a carbapenem antibiotic, along with clindamycin and vancomycin from other classes, all exhibit resistance.

**Table 4:** Antimicrobial susceptibility testing results.

Antimicrobial Agent	Disk Content	C6304	Zone of Inhibition Diameter (Rounded) Breakpoint (mm)			Interpretive Category
		Measured Value	S	I	R	
Ampicillin	10 μg	20.4 ± 0.5	≥17	14-16	≤13	S
Oxacillin	1 μg	0.0 ± 0.0	-	-	-	R
Piperacillin	100 μg	16.2 ± 0.6	≥21	18-20	≤17	R
Cefazolin	30 μg	25 ± 0.5	≥23	20-22	≤19	S
Ceftazidime	30 μg	23.7 ± 0.4	≥21	18-20	≤17	S
Cefalexin	30 μg	21.6 ± 0.2	≥23	20-22	≤19	I
Cefoperazone	75 μg	26.3 ± 0.6	≥21	16-20	≤15	S
Ceftriaxone	30 μg	28.5 ± 0.2	≥23	20-22	≤19	S
Cefuroxime Sodium	30 μg	22 ± 0.5	≥23	15-22	≤14	I
Imipenem	10 μg	18.4 ± 0.8	≥23	20-22	≤19	R
Amikacin	30 μg	22.4 ± 0.5	≥17	15-16	≤14	S
Gentamicin	10 μg	23 ± 0.4	≥15	13-14	≤12	S
Kanamycin	30 μg	22.5 ± 0.4	≥18	14-17	≤13	S
Streptomycin	10 μg	14.9 ± 0.4	≥15	12-14	≤11	S
Doxycycline	30 μg	13.3 ± 0.4	≥14	11-13	≤10	I
Minocycline	30 μg	16.6 ± 0.3	≥16	13-15	≤12	S
Tetracycline	30 μg	14.4 ± 0.6	≥15	12-14	≤11	I
Ciprofloxacin	5 μg	33.7 ± 0.7	≥31	21-30	≤20	S
Levofloxacin	5 μg	29.2 ± 0.6	≥31	21-30	≤20	I
Norfloxacin	10 μg	32.3 ± 0.5	≥17	13-16	≤12	S
Trimethoprim-Sulfamethoxazole	25 μg	16.4 ± 0.6	≥16	11-15	≤10	S
Azithromycin	15 μg	24.2 ± 0.5	≥13		≤12	S
Chloramphenicol	30 μg	27.6 ± 0.5	≥18	13-17	≤12	S
Erythromycin	15 μg	0.0 ± 0.0	-	-	-	R
Clindamycin	2 μg	0.0 ± 0.0	-	-	-	R
Vancomycin	30 μg	0.0 ± 0.0	-	-	-	R

### 3.3 Both SAEO and CEO have inhibitory effects on *Salmonella*, and the combined essential oil composed of them exhibits stronger antibacterial activity

Based on **Table 5**, the MIC of CEO is 0.625 μL/mL, while the MIC of SAEO is 25 μL/mL.As shown in **Table 6**, the FIC of SAEO and CEO was 0.375, possessing synergistic bactericidal effect. The coordination ratio of SCEO was combined the 1/8 MIC (0.078 μL/mL) of CEO with 1/4 MIC (6.25 μL/mL) of SAEO, which as the MIC of SCEO.

**Table 5:** MIC of cinnamon and anise alone against *Salmonella*.

	*Salmonella* (C6304)	*Escherichia coli* (ATCC^®^ 25922™)	*Staphylococcus aureus* (ATCC^®^ 25923™)
CEO	0.62 μL/mL	0.31 μL/mL	0.31 μL/mL
SAEO	25 μL/mL	3.1 μL/mL	12.5 μL/mL
Levofloxacin	0.156 μg/mL	0.078 μg/mL	0.156 μg/mL

**Table 6:** FIC of SAEO combined with CEO against MDR *Salmonella* in MH broth.

Strains	CEO combination/Mono	SAEO Combination/Mono	FIC	functional relationship
MDR *Salmonella*	0.078/0.625	6.25/25	0.375 (<0.5)	collaborate

### 3.4 Essential oils can have a killing effect on *Salmonella* in a very short period of time

**Figure 1:**
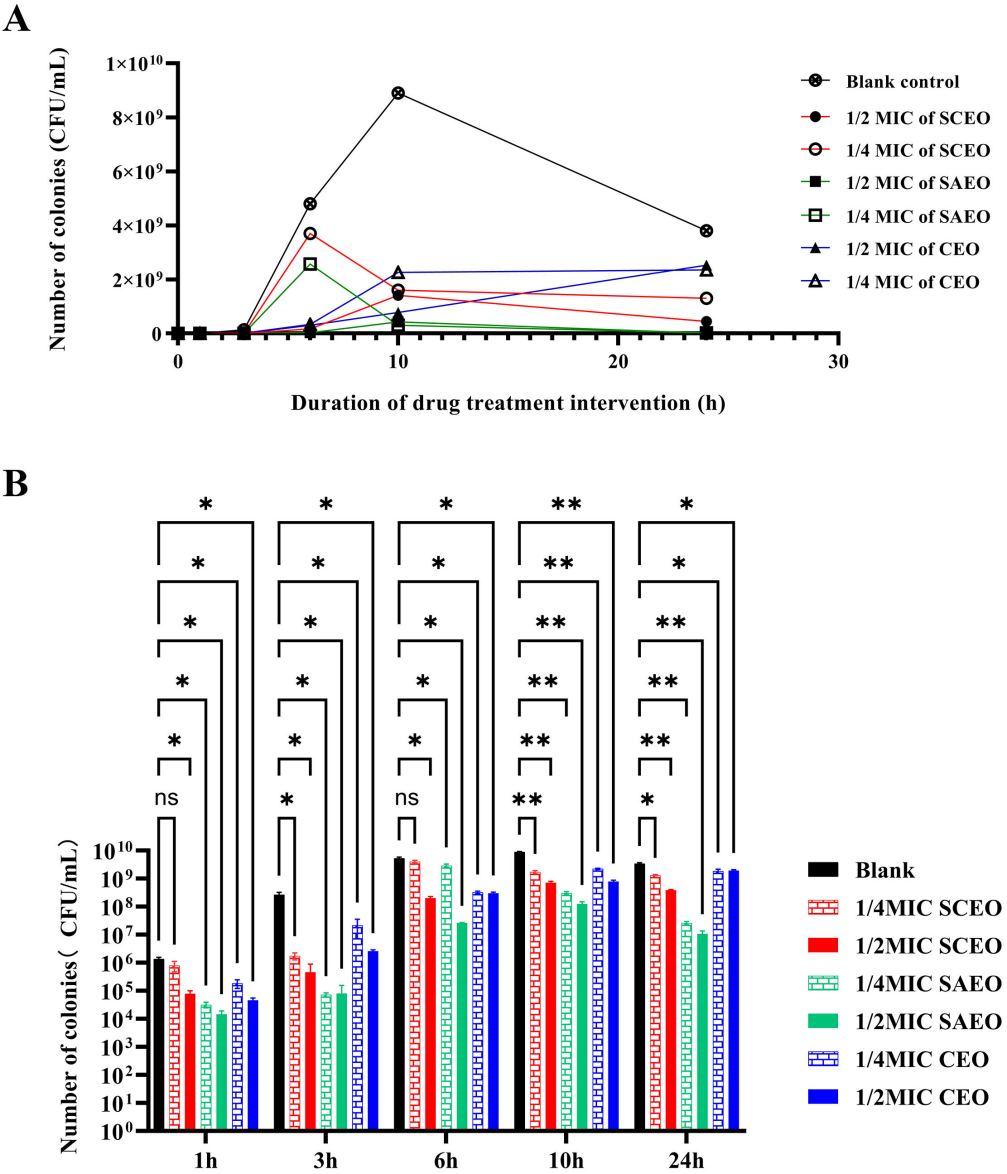
SCEO time-kill curve test. **(A)** Bacterial colony counts at different time points after treatment with various essential oils. **(B)** Bar chart representing the bacterial colony counts at different time points. Compared to the blank group, * indicates p<0.05 (significant difference), ** indicates p<0.01 (highly significant difference), and ns indicates no significant difference.

**Figure 1** explicitly demonstrates the variations in bacterial counts under different treatment conditions. Firstly, the bacterial count in the blank control group significantly increased within 24 hours, which intuitively reflects the natural proliferation rate of bacteria in the absence of drug intervention.

The drug-treated groups exhibited a pronounced bactericidal effect within the initial 6 hours, resulting in a rapid decline in bacterial counts. However, as time progressed, this bactericidal effect gradually diminished, and the rate of bacterial count reduction became more gradual. Despite this, the drug-treated groups were still able to control bacterial proliferation to a certain extent, ensuring that bacterial counts remained at a relatively low level for an extended period.

Notably, the 1/4 MIC SCEO drug group exhibited a relatively weak bactericidal effect in the initial stages, with a small reduction in bacterial counts. However, this does not imply that this drug group is completely ineffective. On the contrary, they were still able to inhibit bacterial growth to a certain extent, maintaining bacterial counts within a relatively stable range (approximately 10^4^ to 10^6^ CFU/mL) at 6 hours. This result indicates that although the CEO drug group exhibited a weaker bactericidal effect in the initial stages, its long-term effect may be limited, yet it still possesses a certain practical value.

### 3.5 Changes in the morphology of bacteria after treatment with essential oils as seen by SEM

**Figure 2:**
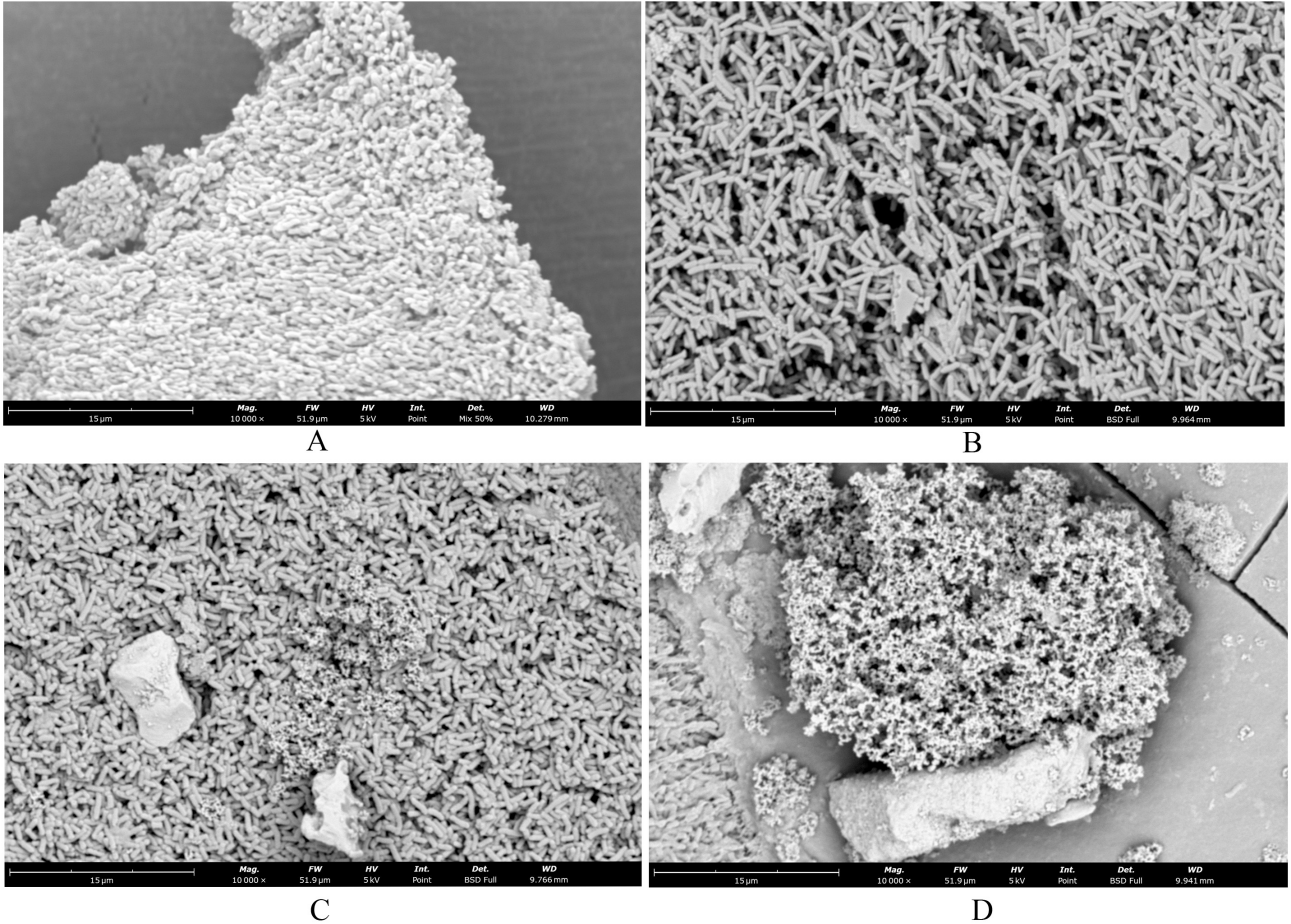
SEM showed changes in cell morphology. **(A)** blank control group 10000×; **(B)** SAEO group 10000×; **(C)** CEO group 10000×; **(D)** SCEO group 10000×.

In the Blank group (**Figure 2A**), the *Salmonella* cells exhibited a short and thick morphology, with a thick layer of membrane-like substances covering their surface, providing protection and stability. After treatment with SAEO (**Figure 2B**), the membrane-like substances on the cell surface almost completely disappeared, resulting in significantly enlarged voids, and the cell morphology transformed into a slender noodle-like shape. Compared to the normal group (**Figure 2C**), although the cells treated with CEO alone showed a tendency of rupture, the reduction in membrane-like substances was not as significant as that observed with SAEO alone. When SAEO was combined with CEO (**Figure 2D**), the membrane-like substances on the cell surface were greatly reduced, and the cells exhibited a distinct rupture morphology, with blurred boundaries between cells and adhesion to each other. This indicates that the active components in the SCEO have a synergistic destructive effect on both the surface and interior of the cells. Additionally, the significant reduction in the number of cells after treatment further confirms the remarkable inhibitory effect of these essential oils on *Salmonella*.

### 3.6 Essential oils reduce *Salmonella* BF formation

**Figure 3:**
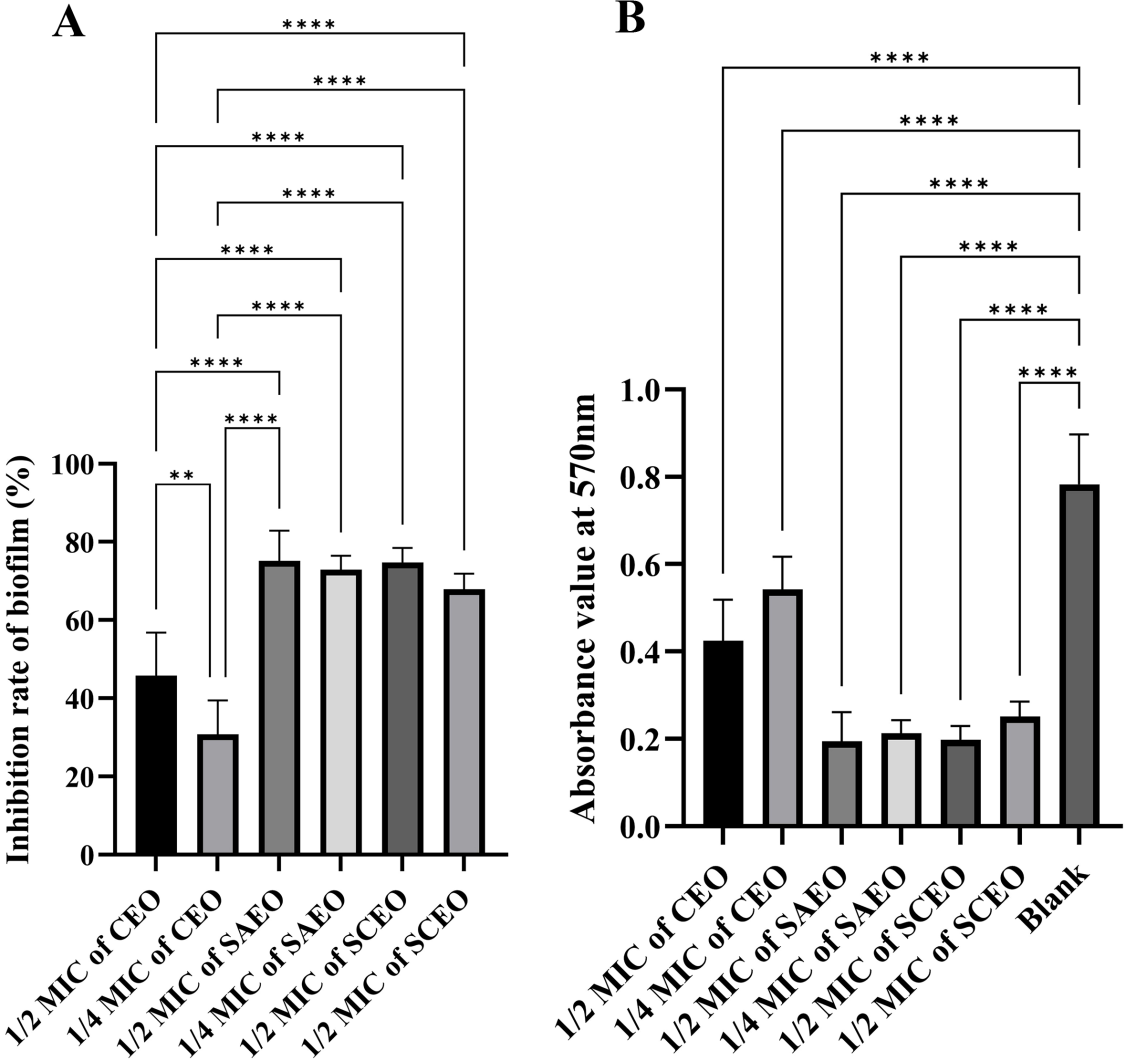
The absorbance value of SCEO on the inhibitory effect of *Salmonella* BF was detected by crystal violet method. **(A)** Inhibition of biofilm formation by different essential oils at various MICs (n=6). The biofilm inhibition rate was calculated using the formula: (Absorbance of control group - Absorbance of drug-treated group)/Absorbance of control group × 100%. The results demonstrate the inhibitory effects of different essential oils on biofilm formation at their respective MICs. *: ** (*p* < 0.01), and **** (*p* < 0.0001). **(B)** This figure presents the raw absorbance data at 570 nm for various drug treatment groups (n=6). *: **** (*p* < 0.0001).

From **Figure 3**, it is observed that there are significant differences in the inhibition rates of *Salmonella* BF formation by SAEO and CEO at different concentrations. Specifically, SAEO and CEO at 1/2 MIC both exhibited inhibition rates of approximately 45% and 75%, indicating that both treatments have a certain effect on inhibiting bacterial BF formation. However, when the concentration was reduced to 1/4 MIC, the inhibition rates generally decreased, with CEO approaching 30% and SAEO also showing a slight reduction. Notably, SCEO at 1/2 MIC (a combination essential oils of 1/8 MIC SAEO and 1/4 MIC CEO) exhibited a significant advantage in inhibition rate, reaching approximately 75%, demonstrating a stronger ability to inhibit bacterial BF formation compared to single-component essential oils. As the drug concentration decreases, the inhibition rate generally shows a downward trend, suggesting that drug concentration is a crucial factor affecting the inhibitory effect.

### 3.7 The SCEO treatment decreased the adhesion of *Salmonella* colonies

**Figure 4:**
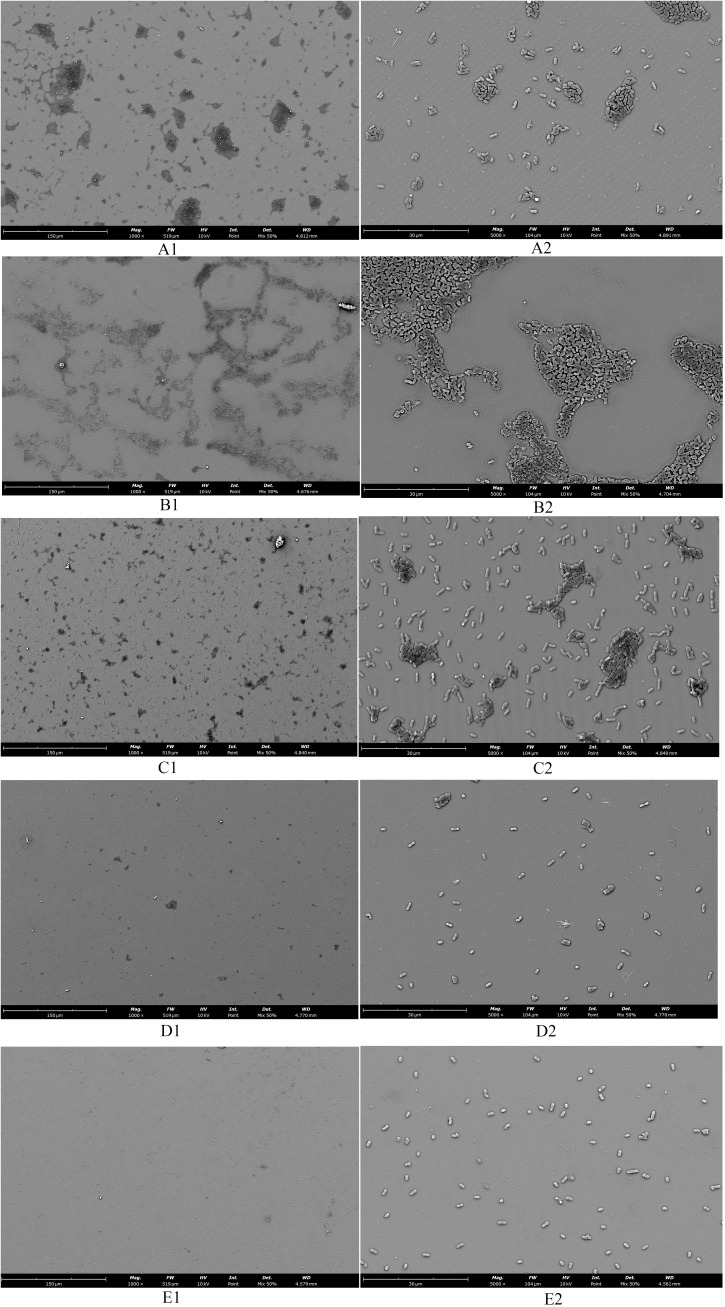
The morphology of adherent-state MDR *Salmonella* organisms treated with the SCEO was detected by the SEM *in vitro*. **(A1)** blank control group 1000×; **(B1)** medium control containing Tween-80 1000×; **(C1)** CEO group 1000×; **(D1)**: SAEO group 1000×; **(E1)** SCEO group 1000×; **(A2)** blank control group 5000×; **(B2)** medium containing Tween-80 control 5000×; **(C2)** CEO group 5000×; **(D2)** SAEO group 5000×; **(E2)** SCEO group 5000×.

**Figure 5:**
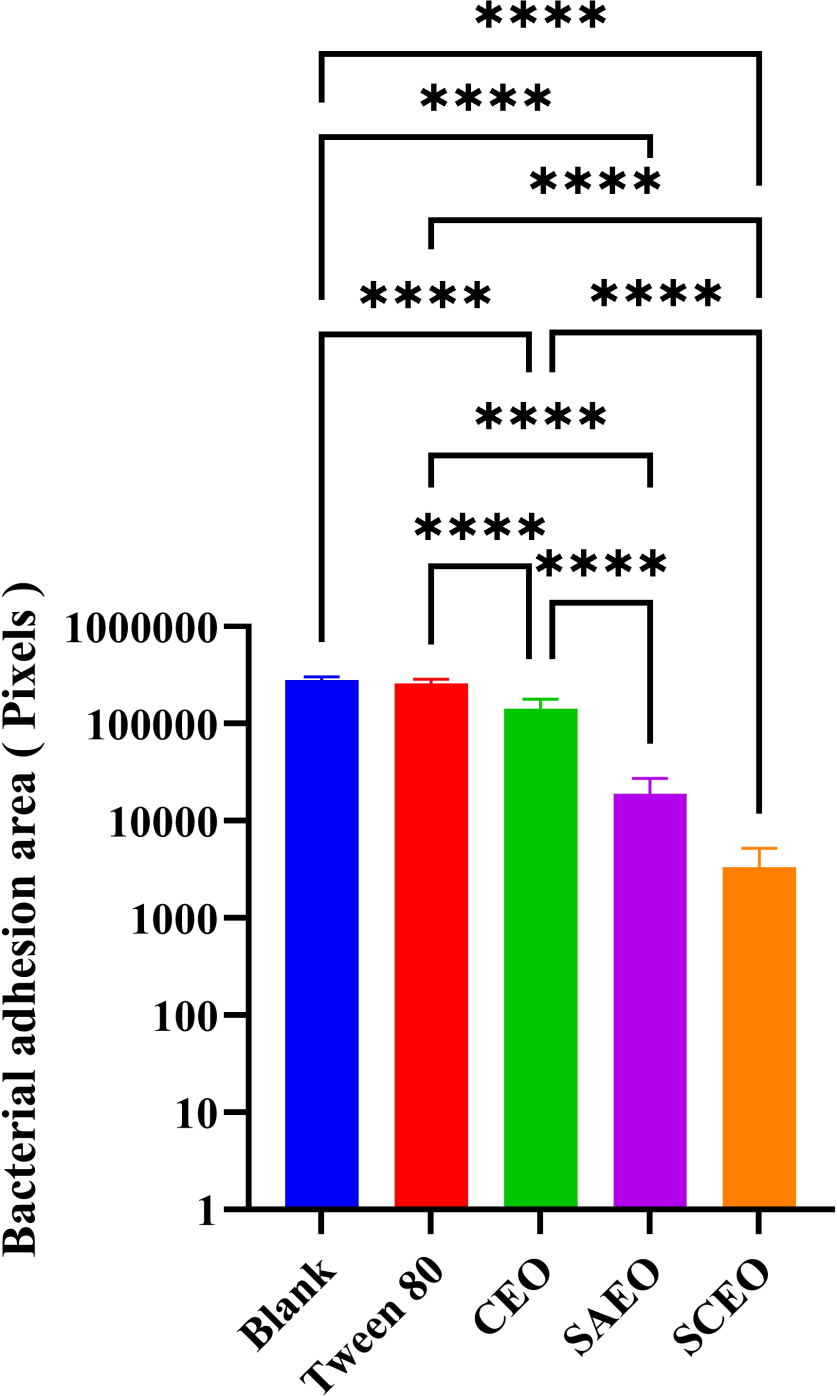
Quantification of bacterial adhesion after drug treatment using SEM. This figure displays the results of a quantitative analysis of bacterial adhesion inhibition following drug treatment, as observed by SEM at 1000× magnification(n = 6). The y-axis of the bar graph is logarithmically scaled to represent the area of bacterial clusters, expressed in pixels. Higher values on the y-axis indicate larger bacterial cluster areas, which correspond to greater bacterial adhesion. The x-axis lists the different drug treatment groups. *: **** (*p* < 0.0001).

Observations of *Salmonella* in the adherent state were conducted using scanning electron microscopy, revealing that SAEO significantly reduced the aggregation behavior of *Salmonella* (**Figure 4D1**). Compared to the blank group (**Figure 4A1**), the number of bacterial colonies present in clusters was significantly decreased. CEO exhibited (**Figure 4C1**) limited inhibitory capacity against this aggregation behavior, with some bacterial clumps still observable under the field of view. Notably, SCEO not only demonstrated an intervention effect on the aggregation behavior of *Salmonella* (**Figure 4E1**) but also had a relatively pronounced disturbance on the bacterial cells. As depicted in **Figures 4E2, A2**, the individual bacterial cells underwent significant shrinkage, suggesting that SCEO may exert an effect on the *Salmonella* cell membrane, thereby preventing the formation of BF. The **Figure 5** reveals no notable discrepancy between the control and Tween groups, implying that the solvent has no impact on *Salmonella* aggregation. In contrast to the control, the CEO group displays a marked difference, indicating CEO’s ability to hinder *Salmonella* aggregation, albeit less effectively than SAEO, which performs notably better. Notably, when CEO is combined with SAEO, SCEO exhibits the most potent inhibition of *Salmonella* aggregation and adhesion at a reduced drug concentration.

### 3.8 SCEO stimulates the expression of biological periplasm-associated mRNAs

**Figure 6:**
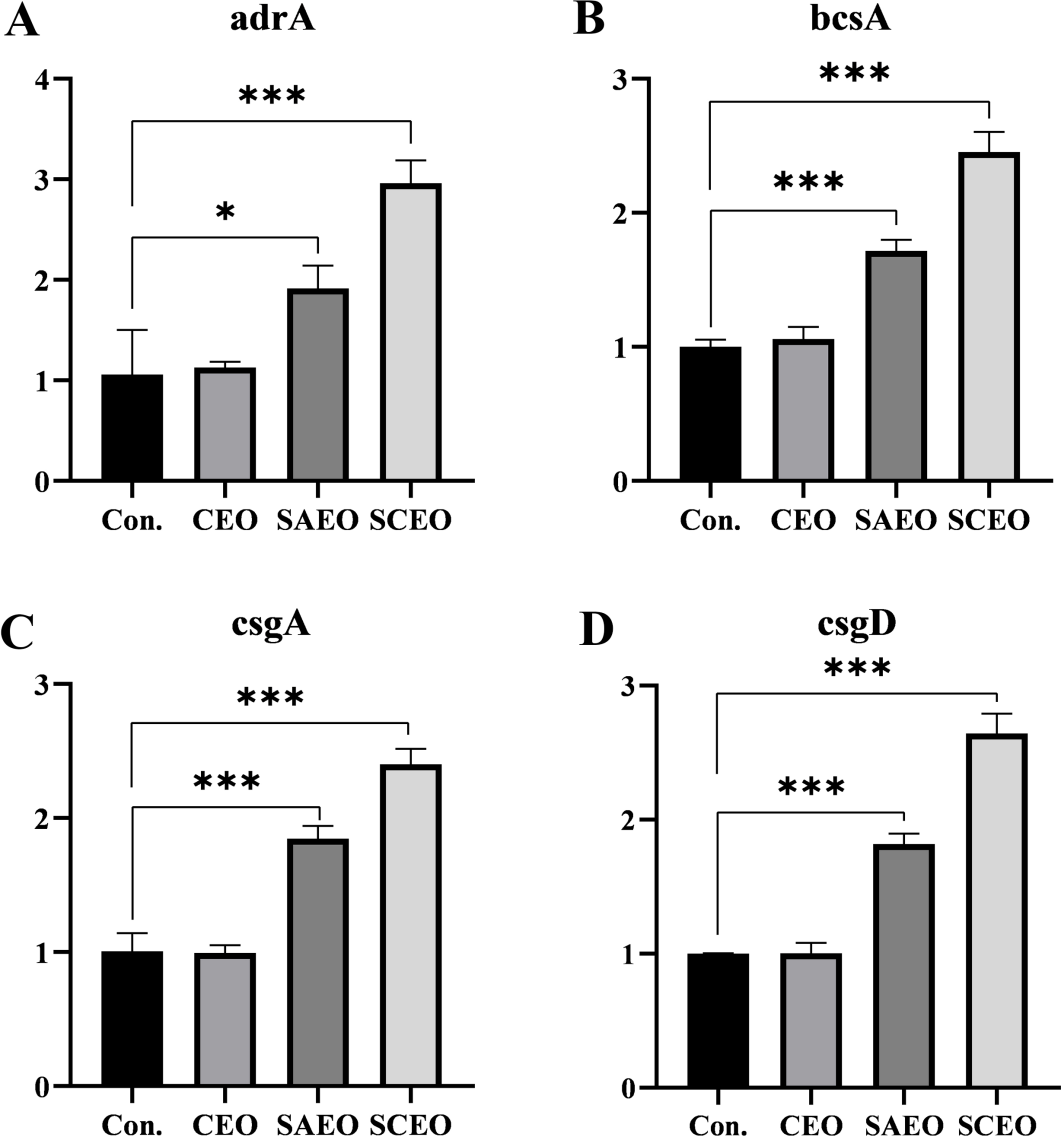
The related genes of BF were detected by the method of qPCR. Figures **(A–D)** represent the gene expression levels of adrA **(A)**, bcsA **(B)**, csgA **(C)**, and csgD **(D)** as measured by qPCR (n=3). *: * (*p* < 0.05), *** (*p* < 0.001).

**Figure 6** provides a detailed illustration of the alterations in the expression of several key BF-related genes (*bcsA*, *adrA*, *csgA*, *csgD*) in *Salmonella* following drug treatment. From the figure, it can be seen that different drug treatment conditions significantly affected gene expression. The CEO treatment group showed no significant changes compared with the blank group, and the SAEO and SCEO treatments significantly up-regulated the expression of *bcsA*, *adrA*, *csgA*, and *csgD* genes. However, this does not imply that the formation of the biofilm was promoted.

### 3.9 The alkaline phosphatase of the *Salmonella* was released by the SCEO

**Figure 7:**
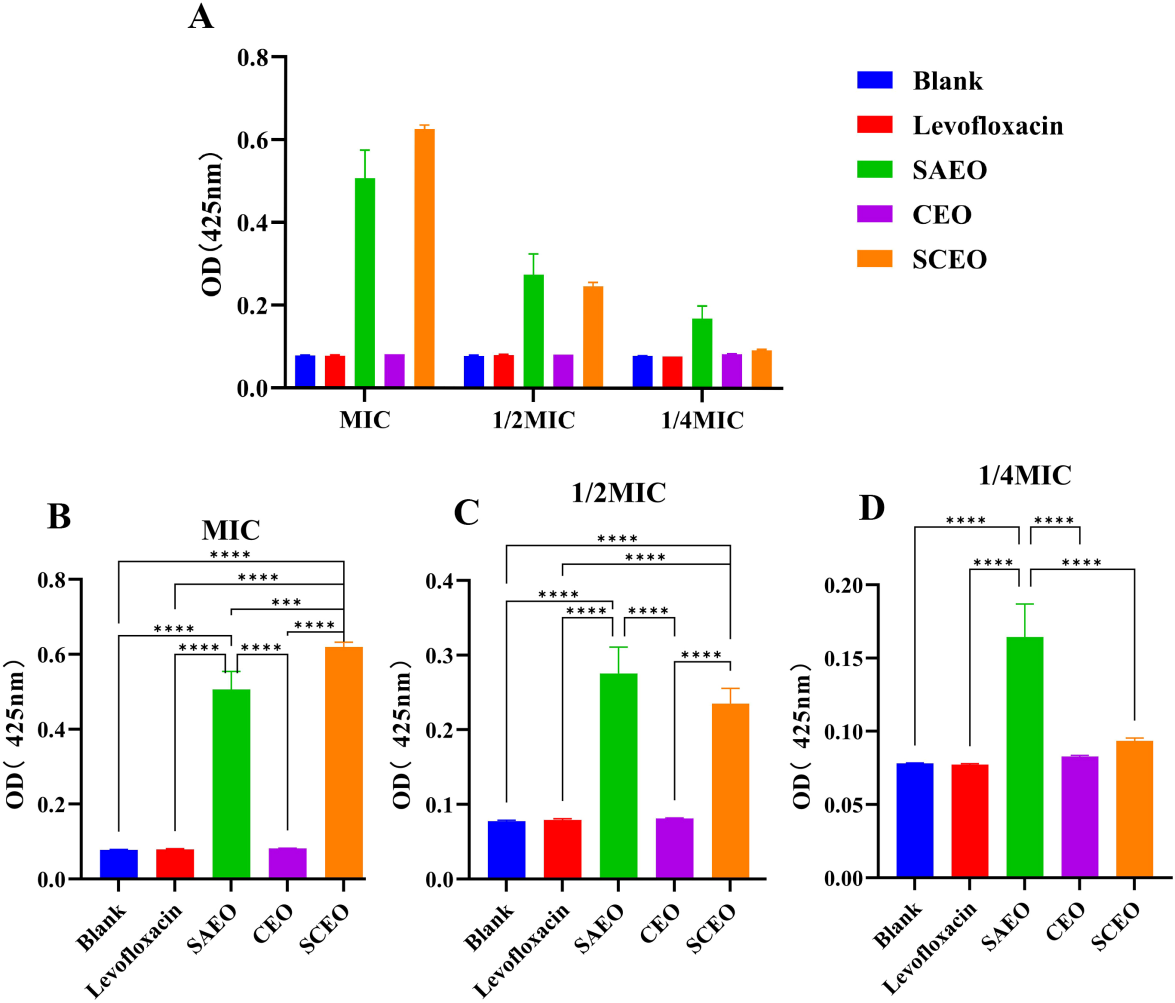
The influence of SCEO on the release of ALP. **(A)** Overview of alkaline phosphatase absorbance values in different treatment groups at three concentration levels: MIC, 1/2MIC, and 1/4MIC. Higher absorbance indicates greater disruption of bacterial cell walls (n=6). **(B–D)** Detailed alkaline phosphatase absorbance values for different treatment groups at specific concentration levels: **(B)** MIC: Alkaline phosphatase absorbance in different treatment groups at 1/4MIC concentration. **(C)** 1/2MIC: Alkaline phosphatase absorbance in different treatment groups at 1/2MIC concentration. **(D)** 1/4MIC: Alkaline phosphatase absorbance in different treatment groups at MIC concentration. *: * (*p* < 0.05), ** (*p* < 0.01), *** (*p* < 0.001)and **** (*p* < 0.0001).

**Figure 7A** demonstrates that at the MIC, both SCEO and SAEO alone can effectively induce the leakage of alkaline phosphatase, indicating their disruptive effects on bacterial cell walls. Notably, SCEO exhibits a more potent disruptive effect than SAEO alone, whereas the disruptive effects of CEO alone or levofloxacin at the same concentration are not evident within 3 hours. **Figure 7B** reveals that at half the MIC (1/2MIC), the disruptive effects of SCEO and SAEO on bacterial cell walls are comparable, with both showing similar efficacy. **Figure 7C** illustrates that when the concentration is reduced to one-quarter of the MIC (1/4MIC), the disruptive effect of SCEO is lower than that of SAEO used alone, highlighting the strong dependence of SCEO’s effect on its concentration. Conversely, SAEO retains some activity at this lower concentration. **Figure 7D** provides an overall view, showing that as the drug concentration decreases, the disruptive effects of both SAEO and SCEO on bacterial cell walls decline. However, the decline in SAEO’s effect is less pronounced than that of SCEO.

Collectively, at the MIC, SCEO exhibits a stronger effect than SAEO or CEO alone, further confirming the synergistic interaction between the components of SCEO (i.e., CEO and SAEO), which produces an effect greater than the sum of their individual effects when combined.

### 3.10 CLSM of SCEO-treated *Salmonella* with live/dead staining

**Figure 8:**
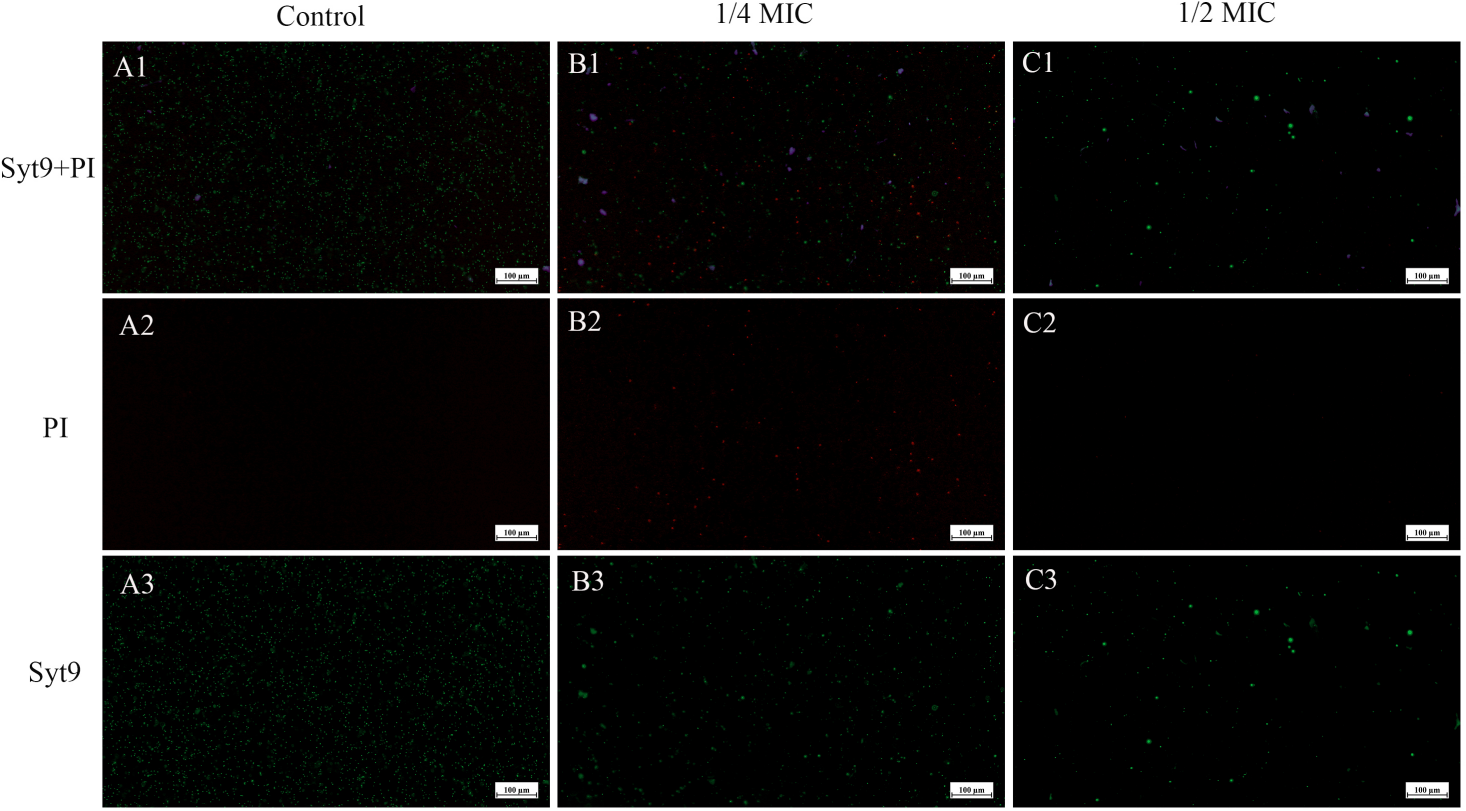
CLSM of SCEO-treated *Salmonella* with live/dead staining. Figure **(A–C)** correspond to the control group, 1/4 MIC, and 1/2 MIC, respectively. Red represents PI staining, and green represents Syto9 staining. Figure **(A1–C1)** show the merged images of PI and Syto9. Figure **(A2–C2)** display the images of PI staining alone, where an increase in the number of bacteria with membrane damage (indicated by PI staining) is observed with increasing SCEO concentrations. Figure **(A3–C3)** show the images of Syto9 staining alone, where a decrease in the number of bacteria with intact cell membranes (indicated by Syto9 staining) is observed as the SCEO concentration increases.

**Figure 8** shows the damage to the bacterial cell membrane after treatment. When the bacterial cell membrane is damaged, PI can penetrate through the membrane’s compromised pores and enter the cell, emitting red fluorescence upon excitation. In contrast, when the cell membrane is intact, PI cannot enter the cell, while Syto 9 can pass through the membrane and emit green fluorescence. This allows for differentiation between live and dead bacteria. From **Figures 8A1-C1**, it is evident that the green fluorescence in A1 covers a large area of the field of view. By comparing A2 and A3, we observe that the bacteria are intact at this stage. After 3 hours of treatment with SCEO at 1/4 MIC, B1 shows a noticeable reduction in green fluorescence spots, along with the appearance of red fluorescence spots, indicating that the bacterial cell membrane is damaged. In C1, after treatment with SCEO at 1/2 MIC, the green fluorescence spots further decrease, and the red fluorescence spots also decrease correspondingly. This might be due to the higher concentration of essential oil disrupting the bacterial integrity, leading to a general reduction in the number of viable cells. It is also worth noting that the green spherical particles in the field of view may represent residual essential oil components that were not completely washed away.

## 4 Discussions

*Salmonella* is an important zoonotic pathogen that poses a continuous threat to global public health due to its multiple modes of infection and the difficulty of prevention and control (Meng et al., 2024). BF not only provides physical and chemical protection for the bacteria, but also enhances their resistance to the host immune response and adverse environmental factors (Chen et al., 2023). It is therefore important to develop novel antibiotic alternatives that can effectively inhibit *Salmonella* BF formation.

In our previous studies, we observed significant differences in the MIC values of SAEO and CEO, both of which were effective at killing Salmonella. This prompted us to explore the potential synergistic effect of their combination. Using the checkerboard method, we confirmed a synergistic interaction between the two oils, which also allowed us to determine the optimal concentration ratio. The results of our study indicated that, when tested against *Salmonella*, our essential oils CEO and SAEO, as well as their combination SCEO, demonstrated significant advantages in inhibiting bacterial growth compared to the six essential oils reported in the literature (Mangalagiri et al., 2021), such as Palm Rosa (MIC 15.625 mg/mL), Lemon Eucalyptus (MIC 31.25 mg/mL), African Marigold (MIC 125 mg/mL), Geranium (MIC 125 mg/mL), Citronella (MIC 250 mg/mL), and Field Mint (MIC 250 mg/mL).

The time-kill curve assay further demonstrated the rapid action of the essential oils, with SCEO eliminating the majority of Salmonella within the first hour (**Figure 1**). Electron microscopy revealed notable morphological changes in the bacteria (**Figure 2**), including elongation of the cells, indicating bacterial damage following treatment.

In the biofilm inhibition assay, crystal violet staining was used to quantify biofilm formation. We found that SCEO, at a much lower concentration (1/4MIC) of 1.56 µL/mL, exhibited effects comparable to or even stronger than SAEO (1/4MIC) at 6.2 µL/mL, supporting the effectiveness of our experimental approach. Additionally, alkaline phosphatase (ALP) leakage was used as an indicator of bacterial cell wall damage (Yu et al., 2023), an enzyme located between the bacterial cell membrane and cell wall, is released into the supernatant when the cell wall is compromised. Using p-nitrophenyl phosphate as a substrate, we were able to quantify this release. The results showed that SCEO, at equivalent concentrations, caused greater bacterial cell wall disruption compared to SAEO.

Based on our research and the existing literature (Zhang et al., 2016, Zhang et al., 2023), these findings suggest several potential mechanisms through which SCEO may exert its bactericidal effects: Firstly, SCEO disrupts the integrity of the bacterial cell membrane (**Figure 8**), leading to a surge in membrane permeability and subsequent leakage of cellular contents. Secondly, active components of SCEO permeate through the bacterial cell wall (**Figure 7**), inhibiting vital protein and DNA synthesis pathways, thereby halting bacterial replication. Thirdly, SCEO impedes essential metabolic pathways, particularly the tricarboxylic acid cycle and hexose monophosphate pathway. Lastly, SCEO induces oxidative stress via the generation of reactive oxygen species (ROS), leading to extensive damage to bacterial cellular components. The collective action of these multifaceted mechanisms enables SCEO to rapidly eradicate planktonic bacteria and sustainably impede their growth.

The ability to eliminate and remove BF from the surface of *Salmonella* is a key consideration in the search for novel and effective biocides. However, currently, there is a scarcity of drugs that can effectively and directly target the formation of these BFs. To date, no BF removal reagents have been commercialized for civilian use, and antimicrobial peptides (Luo et al., 2024) and engineered phages (Szymczak et al., 2024) are the hot topics in anti-BF reagent research.

As can be seen from the experimental **Figure 3**, SCEO has a significant effect on the removal of biological periplasm. Observations of *Salmonella* in the adherent state, conducted using scanning electron microscopy, further corroborate this notion. Notably, SAEO was found to significantly reduce the aggregation behavior of *Salmonella* (**Figures 4D1**, **5**), suggesting its potential as a promising agent for disrupting BF formation.

Although SAEO and SCEO demonstrate similar inhibitory effects on the formation of Salmonella biofilms, it is important to highlight the notable differences observed in their MICs. SAEO has a MIC of 25 µL/mL, whereas CEO has a lower MIC of 0.62 µL/mL. By combining a small fraction (1/80) of SAEO with CEO, we achieved an inhibitory concentration of 6.25 µL/mL for SCEO. This combination allows for a 75% reduction in the amount of SAEO required and an 87.5% reduction in the use of CEO. From a practical and economic perspective, this combination of SAEO and CEO (SCEO) provides several advantages. Not only does it achieve comparable BF inhibition, but it also significantly reduces the overall quantity of essential oils used, lowering both material costs and dependence on a single essential oil. Therefore, SCEO presents a more cost-effective, sustainable solution while maintaining its antibacterial efficacy, which makes it a promising option for future applications.

In our study, we further investigated the effects of the SCEO on the expression of *Salmonella* BF-forming genes. Using quantitative real-time fluorescence PCR (q-PCR), we found significant changes in the expression of key BF-related genes, including *csgA*, *adrA*, *bcsA* and *csgD*, in *Salmonella* exposed to these treatments. Curli amyloid fibers, essential components of BF formed by members of the Enterobacteriaceae family, are closely associated with bacterial adhesion and BF development (Tursi et al., 2020). *CsgD*, a transcriptional activator, orchestrates the synthesis of curli and cellulose in *Escherichia coli* to facilitate this process (Gerstel and Römling, 2003). *CsgD* works in concert with *csgA*, *adrA* and *bcsA* to modulate BF formation either directly or indirectly through their coordinated expression (Ogasawara et al., 2011). In particular, the expression of *csgD* itself is responsive to cellular growth stages and environmental cues. It triggers the biosynthesis of extracellular polymeric matrices consisting of fibrillin, curli and BF-associated proteins (Baps), allowing bacterial cells to transition from a proliferative to a multicellular state (Liu et al., 2014).

We found an interesting phenomenon: While there was no significant change in gene expression in the CEO-treated group compared to the control, SAEO treatment significantly upregulated the expression of *csgD*, *csgA*, *adrA* and *bcsA* (*P*<0.01). Moreover, this upregulation was even more pronounced in the SCEO group compared to SAEO alone. However, this contradiction between the observed increase in gene expression and the significant reduction in BF formation, as indicated by our crystal violet staining results (**Figure 3**), underscores the complexity of bacterial responses to external stressors.

In this context, we offer a nuanced interpretation of the results: As the primary regulator of BF formation, the upregulation of *csgD* in response to stressors such as our experimental treatments may represent an attempt by the bacteria to enhance cell-to-cell adhesion and protect their survival. However, this upregulation may not necessarily result in an increase in BF biomass or structural integrity. Instead, it could be part of a broader stress response, where bacteria activate defensive pathways without necessarily achieving the expected phenotypic outcome (i.e., robust BF formation).Similarly, the increased expression of *csgA*, *adrA*, and *bcsA*—genes essential for BF architecture—may reflect a bacterial stress response rather than a direct enhancement of BF structure. These genes could be upregulated to strengthen the cell wall, alter metabolic pathways, or improve drug resistance, rather than contributing directly to the physical stability of the BF matrix.

From an adaptive evolution standpoint, bacteria may upregulate BF-related genes as a survival strategy when confronted with adverse conditions. By doing so, they aim to create a protective barrier against environmental stressors, including antimicrobials. However, in cases where the treatments are particularly potent or target BF formation mechanisms directly, the bacteria’s attempts to fortify their defenses may prove insufficient to overcome the inhibitory effects.

In conclusion, the upregulation of *csgD* and other BF-related genes in the face of experimental treatments, despite a reduction in BF formation, highlights the intricacies of bacterial stress responses and the multifaceted nature of antimicrobial resistance. Further investigations into the precise mechanisms by which these treatments disrupt BF formation and the bacterial strategies for adaptation will be crucial for the development of more effective antimicrobial strategies.

It’s also worth mentioning that the two types of essential oils studied are entirely sourced from Guangxi, China. As local medicinal herbs that are both food and medicine homologous, this study could provide a new avenue for their development and utilization, potentially driving local economic growth through related industries. Firstly, these essential oils exhibit strong antibacterial properties against Salmonella, a common foodborne pathogen. In the field of food safety, SCEO could serve as a natural and effective food additive to inhibit the growth of Salmonella in meat, dairy products, fruits, and vegetables, thus extending the shelf life of food. Similarly, in animal husbandry, SCEO can be used as a natural feed additive or veterinary medicine to prevent and control Salmonella infections, improving animal health and ultimately protecting human health.

## 5 Conclusions

The SCEO, composed of two essential oils with concentrations of 0.078 mg/mL of CEO and 6.25 mg/mL of SAEO, exhibited enhanced antimicrobial and anti-biofilm effects. Utilizing the alkaline phosphatase method to detect damage to bacterial cell walls, our results further demonstrated that SCEO exerts a detrimental effect on the cell wall structure. Consequently, it is inferred that SCEO reduces the formation of bacterial biofilms by disrupting the integrity of the cell wall, rather than by targeting the csgD gene. Additionally, SCEO can produce a killing effect on Salmonella Thompson through direct contact with the bacterium, displaying a rapid onset of action, and simultaneously inhibiting the production of subsequent Salmonella biofilms.

## Data Availability

The datasets presented in this study can be found in online repositories. The names of the repository/repositories and accession number(s) can be found in the article/**Supplementary Material**.
