# Supplementary material for: Antibacterial and antibiofilm activities of star anise-cinnamon essential oil against multidrug-resistant Salmonella Thompson
Source: Front Cell Infect Microbiol. 2025 Mar 3;14:1463551. doi: 10.3389/fcimb.2024.1463551 (PMC11911814; doi:10.3389/fcimb.2024.1463551)
Supplement: Supplementary file 1 [file Table1.docx]

Supplementary Material

## Supplementary Figures

**Supplementary Figure 1.** The figure legends are required to have the same font as the main text, 12 point normal Times New Roman, single spaced. Please use a single paragraph for each legend and prepare the figures keeping in mind the PDF layout.


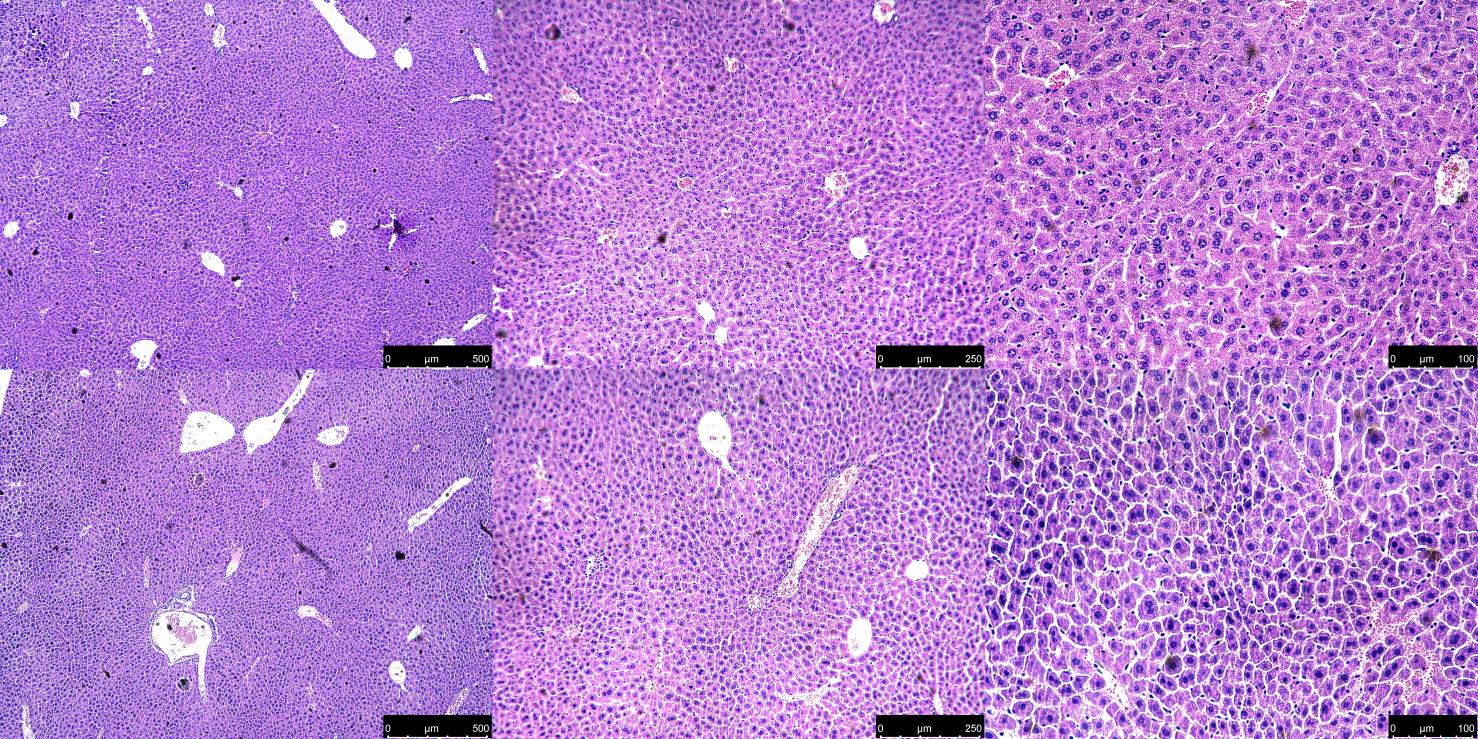


f

e

dc

c

b

a

**Figure S1**. Pathological sections of mouse liver


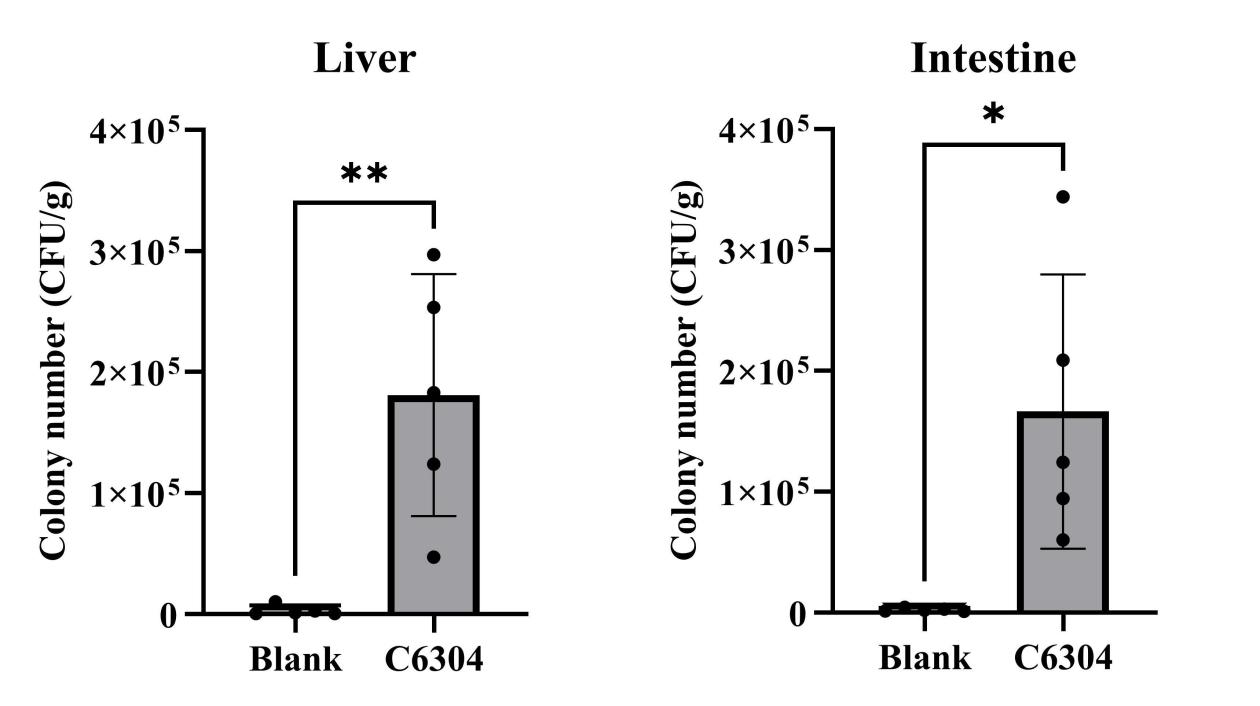


**Figure S2**. Changes in bacterial load in the liver and intestine of mice


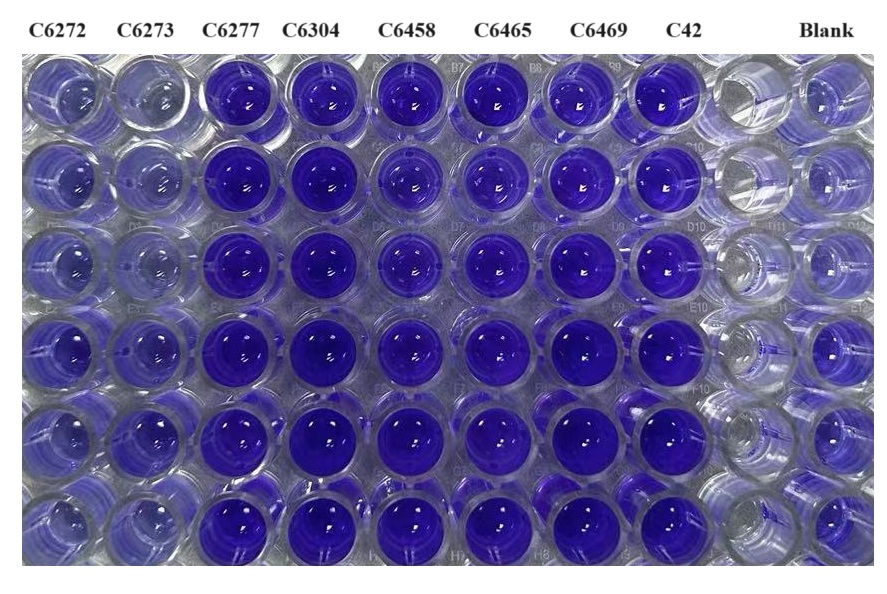


**Figure S3**. Crystalline violet staining of biological periplasm

**Table S1.**Antibiotic susceptibility test quality control results comparison table

| **Antimicrobial Agent** | **Disk Content** | **Escherichia coli**  **(ATCC® 25922™)** | | **Staphylococcus aureus**  **(ATCC® 25923™)** | |
| --- | --- | --- | --- | --- | --- |
|  |  | **Disk Diffusion Method QC Range** | **measured value** | **Disk Diffusion Method QC Range** | **measured value** |
| **Ampicillin** | 10 μg | 15-22 | 18.5±0.3 |  |  |
| **Oxacillin** | 1 μg |  |  | 18-24 | 23.8±0.2 |
| **Piperacillin** | 100 μg | 24-30 | 24.6±0.7 |  |  |
| **Cefazolin** | 30 μg | 21-27 | 24.4±0.5 |  |  |
| **Ceftazidime** | 30 μg | 25-32 | 25.4±0.4 |  |  |
| **Cefalexin** | 30 μg | 15-21 | 18.6±0.2 |  |  |
| **Cefoperazone** | 75 μg | 28-34 | 29.8±0.6 |  |  |
| **Ceftriaxone** | 30 μg | 29-35 | 29.3±0.5 |  |  |
| **Cefuroxime Sodium** | 30 μg | 20-26 | 21.5±0.4 |  |  |
| **Imipenem** | 10 μg | 26-32 | 26.7±0.4 |  |  |
| **Amikacin** | 30 μg | 19-26 | 24.4±0.4 |  |  |
| **Gentamicin** | 10 μg | 19-26 | 24.1±0.9 |  |  |
| **Kanamycin** | 30 μg | 17-25 | 22.1±0.5 |  |  |
| **Streptomycin** | 10 μg | 12-20 | 18.1±0.9 |  |  |
| **Doxycycline** | 30 μg | 18-24 | 18.4±0.9 |  |  |
| **Minocycline** | 30 μg | 19-25 | 20.4±0.6 |  |  |
| **Tetracycline** | 30 μg | 18-25 | 19.8±0.4 |  |  |
| **Ciprofloxacin** | 5 μg | 29-38 | 37.2±0.6 |  |  |
| **Levofloxacin** | 5 μg | 29-37 | 35.6±0.9 |  |  |
| **Norfloxacin** | 10 μg | 28-35 | 34.1±0.6 |  |  |
| **Trimethoprim-Sulfamethoxazole** | 25 μg | 23-29 | 23.5±0.5 |  |  |
| **Azithromycin** | 15 μg |  |  | 21-26 | 21.2±0.2 |
| **Chloramphenicol** | 30 μg | 21-27 | 23.3±0.8 |  |  |
| **Erythromycin** | 15 μg |  |  | 22-30 | 23.7±0.7 |
| **Clindamycin** | 2 μg |  |  | 24-30 | 29.1±0.2 |
| **Trimethoprim-Sulfamethoxazole** | 25 μg | 23-29 | 23.5±0.5 |  |  |
| **Vancomycin** | 30 μg |  |  | 17-21 | 20.6±0.2 |
